# Predicting pain and its association with mortality in patients with stroke

**DOI:** 10.1186/s12883-024-04011-5

**Published:** 2025-01-07

**Authors:** Adam Viktorisson, Aref Haj Hashem, Katharina S Sunnerhagen, Tamar Abzhandadze

**Affiliations:** 1 Department of Clinical Neuroscience, Institute of Neuroscience and Physiology, Sahlgrenska Academy, University of Gothenburg, Gothenburg, Sweden; 2 Department of Rehabilitation Medicine, Sahlgrenska University Hospital, Gothenburg, Sweden; 3 Department of Occupational Therapy and Physiotherapy, Sahlgrenska University Hospital, Gothenburg, Sweden; 4 Department of Clinical Neuroscience, Institute of Neuroscience and Physiology, Sahlgrenska Academy, University of Gothenburg, PO Box 430, Vita Stråket 12, 4th floor, Gothenburg, SE 405 30 Sweden

**Keywords:** Subacute stroke, Predictors, Mortality, Machine learning, Neuropathic pain, Patient Outcome Assessment

## Abstract

**Background and objectives:**

Poststroke pain (PSP) is a prevalent and severe consequence of stroke, encompassing central, neuropathic, and nonneuropathic pain. In this study, we aimed to investigate clinical factors associated with PSP three months after stroke and concurrently explore the association between PSP and one-year mortality.

**Methods:**

This registry-based study comprised data from stroke patients admitted to three hospitals in Sweden between November 2014 and June 2019. The outcome was PSP three months after stroke. Twelve (out of 28) predictor variables were selected by three machine learning methods, and a multivariable binary logistic regression model was fitted for predicting PSP. The association between PSP and one-year poststroke mortality was examined using Cox proportional hazards models.

**Results:**

Among 4,160 stroke patients participating in the three-month follow-up, 54.7% reported PSP. Antiplatelet use, diabetes, hemiparesis, sensory deficits, and need for assistance before stroke were significant predictors of PSP. Male sex, being born in Sweden, higher income, and regular prestroke physical activity predicted the absence of PSP. After adjustment for age, sex, region of birth, and stroke severity, patients experiencing PSP had a significantly higher one-year mortality rate than those without pain, and the most severe level of pain (constant pain) was associated with the highest cumulative mortality.

**Conclusion:**

The study findings indicate treatable factors associated with PSP, which highlight areas of improvement in management strategies. Clinicians should recognize that PSP is associated with increased one-year mortality, emphasizing the importance of pain prevention and treatment for enhanced poststroke outcomes.

**Supplementary Information:**

The online version contains supplementary material available at 10.1186/s12883-024-04011-5.

## Introduction

Pain is a common consequence of stroke. Poststroke pain (PSP) encompasses a diverse range of conditions, usually divided into central neuropathic pain, peripheral neuropathic pain, and nonneuropathic pain [1]. It is estimated that 10–50% of stroke survivors experience some PSP [2–4], of which 70% report pain daily [5]. A recent analysis of pooled clinical trial data also showed that up to 3-9.5% of patients report extreme poststroke pain, with increasing numbers over time [6]. Individuals who experience pain following a stroke often endure other disabling sequelae, including cognitive decline, reduced quality of life, fatigue, and depression [5]. It has also been suggested that patients with PSP are at an increased risk of poststroke mortality [7] and dependency [6]. However, few prior studies have comprehensively evaluated the determinants and outcomes of PSP. Moreover, consensus guidelines for the management of PSP are lacking.

Factors such as female sex, severe stroke symptoms, functional dependency, fatigue, excessive alcohol use, diabetes mellitus, depression, antithrombotic treatment, and statin use have previously been associated with a higher probability of PSP [2,7–12]. These associations have, however, not been consistent across studies. Understanding modifiable factors contributing to PSP is an important step in informing the development of effective management strategies and guidelines. Understanding the consequences of PSP is necessary for targeted interventions.

The objective of this study was to investigate clinical factors associated with PSP three months after stroke and explore the association between PSP and one-year mortality. As many factors may be associated with PSP, we use supervised machine learning methods to identify the set of variables that could have importance in PSP. Machine learning techniques have gained prominence in medical research due to their ability to manage large, complex datasets and identify intricate patterns that may not be apparent using traditional statistical methods. In addition, we assumed our approach to be particularly advantageous when dealing with the vast amount of routinely collected healthcare data often available in stroke cohorts^13,]^ as traditional statistical methods may struggle to handle complex interactions and patterns of multifactorial outcomes such as pain, whereas machine learning algorithms excel in extracting meaningful insights [14,15]. 

## Methods

The Strengthening the Reporting of Observational Studies in Epidemiology (STROBE) Statement was followed [16].

### Study design

The study sample consisted of patients admitted to the stroke units at three hospitals in Gothenburg, Sweden between November 1, 2014, and June 30, 2019, and included in the local stoke register (Väststroke). All stroke diagnoses were clinically verified, and all patients underwent brain imaging to differentiate between ischemic stroke and intracerebral hemorrhage. Additional patient in-hospital characteristics were collected from medical records and the national Swedish Stroke Register (Riksstroke). All patients were invited to participate in a three-month postal follow-up facilitated by Riksstroke. Information on prior strokes and prestroke medications was also obtained from Riksstroke. Socioeconomic data were collected from the Integrated Database for Health Insurance and Labor Market Studies. Present comorbid conditions were identified in the National Patient Register. Mortality rates and causes of death were obtained from the Cause of Death Register. The data were merged and pseudonymized by the National Board of Health and Welfare.

### Outcomes and covariates

The primary outcome of the study was PSP reported at three months after stroke. To evaluate pain, each participant was asked to rate their present pain intensity using a four-tier scale, encompassing the following categories: absence of pain, occasional pain, regular pain, or persistent pain. In regression analyses, the PSP was dichotomized into two groups: those reporting no pain and those experiencing pain, which included the remaining three categories. In addition, one-year mortality rates were monitored for each patient who participated in the three-month follow-up. Both stroke-related deaths and deaths from other causes were recorded.

Covariates were collected at baseline, which was defined as the time of incident stroke for each patient. Region of birth was classified as native-born in Sweden or abroad. Educational level was categorized as having a postsecondary education (> 12 years). Income was measured based on household income in the year prior to the stroke, and a high income was defined as an income within the highest tertile of the total cohort. Single households were defined as patients living alone without any other family members or unrelated individuals in the same dwelling. Smoking status was classified as either current smoking or absence of smoking for less than one year. Alcohol abuse was defined according to relevant conditions using the International Classification of Diseases 10th revision (ICD-10). Prestroke physical activity was assessed using the Saltin-Grimby Physical Activity Level Scale (SGPALS) and divided into three levels: sedentary, light physical activity, and moderate to high physical activity [17]. Information on prestroke medications was obtained for antihypertensive, antiplatelet, anticoagulant, and lipid-lowering drugs. Comorbid conditions were defined according to the ICD-10 and included atrial fibrillation, cancer, depression, diabetes, chronic obstructive pulmonary disease (COPD), renal failure, hyperlipidemia, and prior strokes. Interventional stroke care was recorded, including a range of neurosurgical procedures (hemicraniectomy, cerebrospinal fluid drainage, and hematoma evacuation) as well as thrombectomy and intravenous thrombolysis. Stroke severity at admittance was evaluated using the National Institutes of Health Stroke Scale (NIHSS) [18]. Stroke sequelae, including hemiparesis and sensory deficits, were recorded if present during the hospital stay. The Montreal Cognitive Assessment (MoCA) was used to assess impairments in cognitive function at the stroke unit. The total possible score on the MoCA is 30 points, and a score of ≥ 26 was considered normal cognition [19]. The patients’ need for assistance was also assessed, distinguishing between those who were independent in their activities of daily living and those who needed help from others.

## Statistics

Patients who reported intermittent, frequent, and constant pain were combined into a single group for regression analyses. Drop-out analyses were conducted using the χ² test for categorical variables and the Mann‒Whitney U test for continuous variables. Univariable binary logistic regression was used to evaluate the associations between covariates and PSP at three months. We calculated Cramer’s V coefficients to assess multicollinearity among covariates. Correlation coefficients ≥ ± 0.7 were set as a threshold for multicollinearity. The analyses were performed in R version 4.0.5 and Python version 3 [20]. All statistical tests were two-tailed at an alpha level of 5%.

### Missing data

A missing data matrix was constructed to visualize the distribution of missing data, with no clear patterns of missingness across different observations (Supplementary Fig. 1). Multiple imputation by chained equations (MICE) was used to handle missing observations prior to the machine learning procedure [21]. The MICE algorithm imputes missing values by creating multiple sets of plausible values based on observed data and relationships among all other variables. We created five imputed datasets, and each imputation underwent 20 iterations. The predictive mean matching method was employed, and a minimum correlation of 0.1 was considered in the prediction models. All included covariates fulfilled the requirement of at least 80% useful data. The imputed data were evaluated using density plots, which show that imputed values follow the distributions of the actual data (Supplementary Fig. 2).

### Variable selection

To identify a parsimonious model with enhanced generalizability, we employed a variable selection approach by developing three distinct supervised machine learning models. Covariates (*n* = 28) included in the analyses were based on previous literature [2,7–12] and availability of the data in the registries. The imputed data were split into training and testing sets, with 80% of the data used for training and 20% for testing. We employed binary classifiers least absolute shrinkage and selection operator (LASSO, logistic regression with L1 penalty [22], random forests [23], and eXtreme Gradient Boosting (XGBoost) [24] due to their different natures. LASSO is an ML model with regularization. Both random forests and gradient boosting are ensemble methods, but they differ in their construction approach and how they handle errors during training. Each machine learning model was fitted, and the hyperparameters were tuned in the training dataset by using 5-fold cross validation. The models were evaluated in testing datasets by obtaining the receiver operating characteristic curve (AUC-ROC).

1. LASSO logistic regression minimizes logistic loss with an L1 penalty on coefficients, promoting feature selection [22]. It is useful for high-dimensional datasets, offering control over sparsity and mitigating overfitting through a regularization hyperparameter (λ or alpha) [22].
2. Random forest: This is an ensemble learning method that constructs multiple decision tree models during training and combines their predictions to make final predictions. Each decision tree is built on a random subset of the data and a random subset of the features. This randomness helps to reduce overfitting and improve the model’s generalization ability. In binary classification tasks, each decision tree in the forest predicts the probability of the positive class, and the final prediction is obtained by averaging these probabilities across all trees [23]. Cross-validation was used to find the optimal number of trees and features considered at each split.
3. XGBoost is a powerful machine learning algorithm known for its efficiency and high performance [24]. It belongs to the boosting family and works by combining the predictions of multiple weak models, typically decision trees. XGBoost employs a gradient boosting framework, optimizing both accuracy and computational speed [24].

Variable importance values were obtained for each algorithm. The variable was regarded as an important predictor for PSP if it was selected by all three ML methods, in accordance with the methodology previously described by Mostafaei et al. [25]. The threshold for coefficient value was set at > 0.01.

Prediction of PSP:

A multivariable binary logistic regression model was fitted in the complete dataset for predicting PSP 3 months after stroke. Twelve variables identified as important from the variable selection step were included in the regression model. At the variable level, we obtained Odds Ratios (OR), 95% confidence intervals (CIs) and P values. The regression model was evaluated with AUC-ROC and Akaike information criteria (AIC).

### Survival analysis

Cumulative one-year mortality following the three-month follow-up was described using Kaplan‒Meier curves. Hazard ratios (HRs) and 95% CIs for one-year mortality were obtained from three Cox proportional hazards models. Model 1 represented the crude association between PSP and one-year mortality. Model 2 was adjusted for age, sex, and region of birth. Model 3 was adjusted for age, sex, region, and stroke severity measured by NIHSS. As NIHSS had missing observations (*n* = 221), model 3 was constructed using both the original and imputed data. Schoenfeld residuals were utilized to test the independence between residuals and time. All variables were evaluated as potential confounding factors in the Cox proportional hazards model. However, additional covariate adjustments violated the proportional hazards assumption, as determined by global correlations between scaled Schoenfeld residuals and time.

## Results

### Study sample

There were 6,491 patients treated for stroke during the study period. Among them, 4,160 (64%) participated in the three-month follow-up, 1051 (16%) had died, and 1,280 (20%) did not respond. The frequency of missing observations and drop-out analyses are available in Supplementary Table 1. Included patients were younger and less affected by their stroke with a lower proportion of impaired cognition and need of assistance, although the largest difference was observed between included and deceased patients. Of 4,160 patients, 1,844 (44.3%) reported no pain, 1,214 (29.2%) reported intermittent pain, 642 (15.4%) reported frequent pain, 369 (8.9%) reported constant pain, and 91 (2.2%) were unsure about their pain status (excluded from analyses). In total, 2,225 (54.7%) of 4,069 patients reported PSP. Table 1 presents the baseline characteristics of the included patients. Unadjusted odds ratios of PSP at the three-month follow-up are presented in Table 1.

**Table 1:** Baseline characteristics and associations between covariates and poststroke pain, no. Of patients 4069

	Baseline characteristics		Unadjusted associations	
	No PSP (*n* = 1844)	PSP (*n* = 2225)	Odds ratio (95% CI)	*p* value *
Age, mean (SD)	72 (13)	74 (13)	1.01 (1.00-1.02)	< 0.001
Male sex	1139 (62)	1038 (47)	0.54 (0.48–0.61)	< 0.001
Born in Sweden	1611 (87)	1734 (78)	0.51 (0.43–0.60)	< 0.001
Education > 12 years	515 (28)	515 (24)	0.78 (0.68–0.90)	< 0.001
High income	816 (44)	695 (31)	0.57 (0.50–0.65)	< 0.001
Single household	767 (42)	1039 (47)	1.25 (1.10–1.41)	< 0.001
Smoking	193 (12)	292 (15)	1.27 (1.04–1.54)	0.018
Alcohol abuse	31 (2)	72 (3)	1.96 (1.29–3.03)	0.002
Prestroke physical activity				
Sedentary	701 (42)	1133 (56)	ref	-
Light intensity	801 (48)	774 (39)	0.60 (1.47–1.78)	< 0.001
Moderate or high intensity	164 (10)	99 (5)	0.37 (0.29–0.49)	< 0.001
Prestroke medications				
Antihypertensives	973 (53)	1264 (57)	1.18 (1.04–1.34)	0.009
Antiplatelets	424 (23)	603 (27)	1.24 (1.08–1.44)	0.003
Anticoagulants	212 (12)	256 (12)	0.99 (0.82–1.21)	0.947
Statins	468 (25)	641 (29)	1.19 (1.03–1.37)	0.014
Comorbidities				
Atrial fibrillation	386 (24)	491 (24)	1.04 (0.89–1.21)	0.645
Cancer	186 (10)	257 (12)	1.16 (0.95–1.42)	0.136
Depression	65 (4)	144 (7)	1.89 (1.41–2.57)	< 0.001
Diabetes	302 (16)	476 (21)	1.39 (1.19–1.63)	< 0.001
COPD	101 (6)	216 (10)	1.86 (1.45–2.37)	< 0.001
Renal failure	127 (7)	165 (7)	1.08 (0.85–1.38)	0.516
Hyperlipidemia	278 (15)	392 (18)	1.20 (1.02–1.42)	0.030
Prior stroke	255 (14)	359 (16)	1.20 (1.01–1.43)	0.038
Stroke type				
Ischemic stroke	1677 (91)	1999 (90)	ref	-
Intracerebral hemorrhage	163 (9)	222 (10)	1.14 (0.92–1.41)	0.219
Neuro interventional therapy ^a^				
Neurosurgery	4 (0.2)	11 (0.5)	0.90 (0.77–1.05)	0.184
Thrombectomy	212 (12)	241 (12)	-	
Thrombolysis	298 (16)	305 (14)	-	
NIHSS, mean (SD)	4 (5)	5 (6)	1.03 (1.02–1.04)	< 0.001
Hemiparesis	450 (28)	700 (38)	1.53 (1.32–1.76)	< 0.001
Sensory deficit	348 (22)	562 (31)	1.56 (1.33–1.82)	< 0.001
Cognition ^b^				
Normal	356 (24)	337 (19)	ref	-
Impaired	563 (38)	649 (37)	1.22 (1.01–1.47)	0.039
Unclear	573 (38)	794 (45)	1.46 (1.22–1.76)	< 0.001
Need of assistance before stroke	221 (13)	432 (20)	1.76 (1.48–2.10)	< 0.001

### Variable selection

**Fig. 1:**
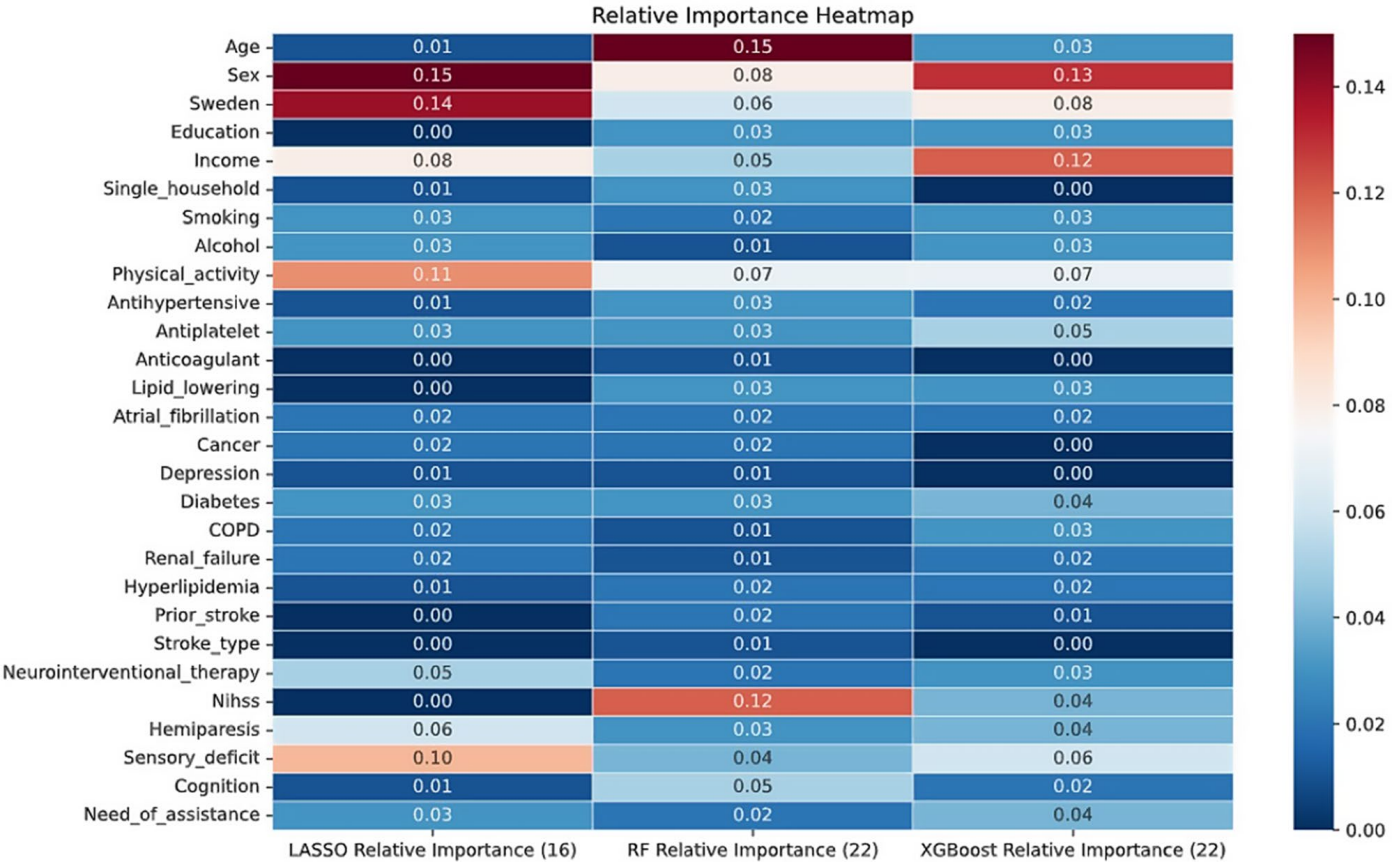
Relative variable importance values for LASSO, random forests, and extreme gradient boosting models (results based on the training dataset 80%, No. of patients 3300); higher absolute values indicate a stronger impact. The numbers in parentheses indicate the count of variables with importance values greater than 0.01. A total of 12 variables were selected by the machine learning method. The AUC-ROC (95% CI) values for LASSO, random forest, and XGBoost were 0.65 (0.61–0.69), 0.64 (0.61–0.68), and 0.64 (0.61–0.68), respectively (results based on the test dataset 20%, No. of patients 814)

The LASSO, random forest, and XGBoost models identified 16, 22, and 22 variables, respectively (Fig. 1). The corresponding AUC-ROC (95% CI) values for LASSO, random forest, and XGBoost were 0.65 (0.61–0.69), 0.64 (0.61–0.68), and 0.64 (0.61–0.68), respectively. All three models shared 12 variables with coefficients exceeding 0.01.

### Prediction of poststroke pain

The results of the multivariable binary logistic regression model are presented in Table 2. The ORs (range 0.55–0.80) of PSP 3 months after stroke decreased if the patient was male, born in Sweden, had high income, had light physical activity, had moderate to high physical activity, or had neurointerventional therapy. However, the ORs (range 1.18–1.50) of PSP 3 months after stroke increased if the patient used antiplatelets, had diabetes, had hemiparesis, had sensory deficits, or needed assistance before stroke.

**Table 2:** Results of the multivariable binary logistic regression model predicting poststroke pain three months after stroke, no. Of patients 4069

Variables	OR	95% CI		*P* values
Male sex	0.60	0.53	0.69	< 0.001
Born in Sweden	0.55	0.46	0.66	< 0.001
High income	0.78	0.68	0.89	< 0.001
Smoking	1.15	0.95	1.40	0.14
Light intensity PA	0.73	0.63	0.84	< 0.001
Moderate to high intensity PA	0.56	0.43	0.74	< 0.001
Antiplatelet use	1.18	1.01	1.37	0.04
Atrial fibrillation	0.90	0.77	1.05	0.18
Diabetes	1.25	1.06	1.48	< 0.05
Neuro interventional therapy	0.80	0.67	0.96	< 0.05
Hemiparesis	1.26	1.08	1.48	< 0.01
Sensory deficit	1.50	1.27	1.77	< 0.001
Need of assistance before stroke	1.27	1.05	1.54	< 0.05

Note: All variables represent the presence of the condition or characteristic

PA, Physical activity before stroke. Odds ratios and 95% confidence intervals (CI) were calculated using a multivariable binary logistic regression model. AUC (95% CI), 0.65 (0.64–0.68). Akaike information criteria, 5315

### Survival analyses

**Fig. 2:**
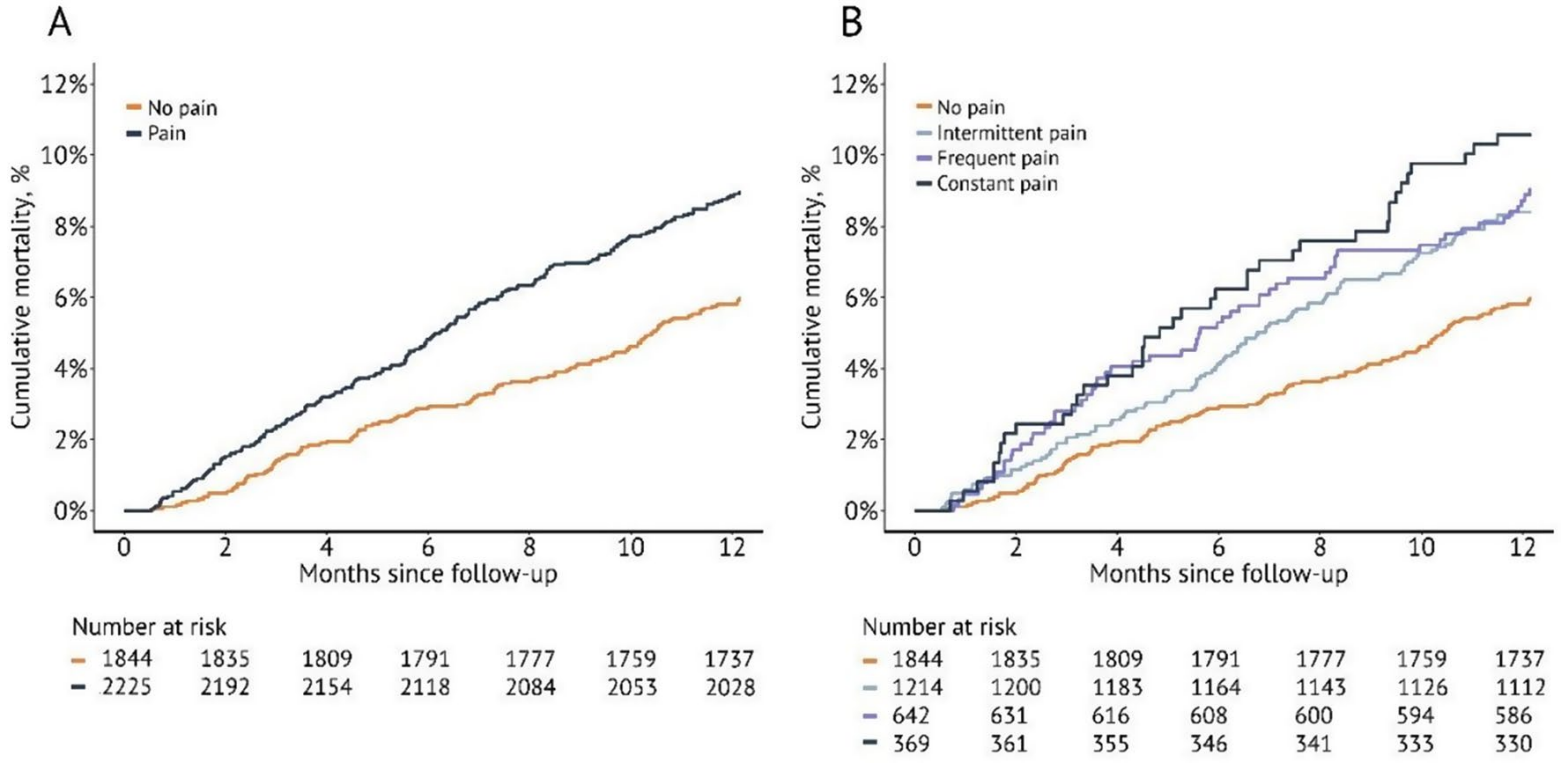
Cumulative one-year mortality stratified by poststroke pain. Kaplan‒Meier curves for cumulative one-year mortality after the three-month follow-up stratified by (**A**) pain or no pain and (**B**) all available responses

One year after the three-month follow-up, 199 (8.9%) patients with PSP had died compared to 110 (6.0%) patients with no pain. The cumulative mortality stratified by PSP is presented in Fig. 2A. Patients with constant pain at three months had the highest cumulative mortality (Fig. 2B). The frequency of stroke as a cause of death was similar between patients with and without PSP (43 of 199 [21.6%] versus 22 of 110 [20.0%]). Hazard ratios of mortality within one year after the three-month follow-up are presented in Table 2. In the unadjusted model, patients with PSP were significantly more likely to die within one year (HR 1.55, 95% CI 1.22–1.96) than patients without PSP. After adjusting for age, sex, and region of birth, the hazard ratio remained significantly increased (HR 1.45, 95% CI 1.14–1.84). Moreover, even when accounting for stroke severity in the analysis, the association between PSP and increased mortality persisted using the original nonimputed dataset (HR 1.38, 95% CI 1.08–1.78). This finding was further confirmed by similar results obtained from the imputed dataset (HR 1.37, 95% CI 1.08–1.73), Table 3.

**Table 3:** Associations between poststroke pain and one-year mortality

		No. of patients	HR (95% CI)	*p* value *
Model 1	Poststroke pain *unadjusted*	4069	1.55 (1.22–1.96)	**< 0.001**
Model 2	Poststroke pain *adjusted for age*,* sex and region*	4069	1.45 (1.14–1.84)	**0.002**
Model 3	Poststroke pain *adjusted for age*,* sex*,* region*,* and stroke severity*	3848	1.38 (1.08–1.78)	**0.010**
		4069 (imp. data)	1.37 (1.08–1.73)	**0.009**

* Bold text indicates significance (*p* < 0.05). Hazard ratios and 95% confidence intervals were calculated using Cox proportional hazard models. Model 1 represented the crude association between poststroke pain and one-year mortality. Model 2 was adjusted for age, sex, and region of birth. Model 3 was adjusted for age, sex, region of birth and stroke severity measured by National Institutes of Health Stroke Scale scores (missing observations *n* = 221). Global correlation between scaled Schoenfeld residuals and time: Model 1 χ²=1.89, *p* = 0.17; Model 2 χ²=7.59, *p* = 0.108; Model 3 χ²=9.08, *p* = 0.106.

## Discussion

This study aimed to explore factors associated with poststroke pain (PSP) three months after stroke, utilizing multidimensional clinical data and a supervised machine learning approach. Additionally, the relationship between PSP and one-year survival following the three-month assessment was investigated. Notable predictors of PSP included sociodemographic features, stroke-related deficits, prestroke conditions, and comorbidities. The findings highlight the intricate interplay of factors contributing to PSP, encompassing sociodemographic traits, preexisting conditions, lifestyle choices, and stroke-related neurological impairments. Furthermore, patients experiencing PSP exhibited a significantly higher one-year mortality rate than those without pain.

We found that sex was a significant predictor of PSP. This finding is in line with previous studies, indicating that males have lower odds than females [3,26] Likewise, stroke sequelae in terms of sensory deficits and hemiparesis have previously been shown to be associated with PSP [9,10]. On the other hand, factors such as region of birth and prestroke physical activity emerged as novel predictors of PSP. These findings highlight the importance of considering sociodemographic and lifestyle factors in understanding poststroke pain. Age did not show a significant association with PSP. Prior studies have reported conflicting findings regarding the relationship between age and PSP. One study found that increased age was associated with PSP [10], while others found the opposite [7,12]. The variability in these results highlights the complexity of factors influencing PSP and suggests that age alone may not be a decisive predictor for its occurrence.

We found that the need for assistance in daily activities was significantly associated with PSP. This is in line with previous studies showing positive associations between limitations in activity capacity and mobility [2,10]. However, we cannot conclude a causal association between activity and mobility limitations and PSP. Furthermore, physical activity prior to stroke also emerged as a significant predictor of PSP. Both light intensity physical activity and moderate to high physical activity were associated with the absence of pain. The association was stronger between regular physical activity and the absence of pain compared to light physical activity, which indicates that there may be a dose‒response to this association. Although no prior study has explored the association between prestroke physical activity and poststroke pain, our finding is consistent with previous research that has demonstrated the effectiveness of physical activity on chronic pain [27–29]. 

Prior studies have used several different time points for the evaluation of PSP, ranging from the acute phase to 5 years poststroke [2,7–12]. The early subacute phase of stroke recovery, extending up to three months, involves neural repair and adaptation, where functional improvements are most prominent [30]. By three months, neurological recovery tends to stabilize, as compensatory mechanisms peak, and residual deficits become more apparent. Pain, having interacted with these recovery processes, may have reached a relatively stable state by this point, making the three-month mark suitable for evaluating its presence and impact on poststroke outcomes. The three-month poststroke evaluation of pain also captured both early-onset and persistent pain, facilitating clinical relevance and the feasibility of interventions.

After the three-month follow-up, we found an association between PSP and one-year mortality. Patients with PSP had a significantly higher risk of mortality than those without pain. This finding aligns with prior research suggesting that pain after stroke may have adverse effects on patient outcomes and overall survival [7,31]. The association between PSP and mortality persisted even after adjusting for age, sex, region, and stroke severity, indicating that poststroke pain may independently contribute to a higher risk of mortality. All patients with PSP faced a significantly higher risk of mortality than those without pain, and constant pain was associated with the highest cumulative mortality in the following year.

Using a range of supervised machine learning approaches in data analysis generally offers many advantages. First, it allows for the identification of complex patterns and interactions among multiple variables often present in stroke cohorts, which might not be captured using traditional statistical methods [13,32]. The machine learning models used in this study were capable of handling large numbers of predictors and could provide insights into the relative importance of each variable in predicting the outcome. Second, machine learning approaches were less prone to assumptions about the underlying data distribution, making them more robust and adaptable to different types of datasets [33]. This was particularly valuable in studies with diverse and multidimensional data, where relationships between predictors and outcomes may be nonlinear and complex. However, the classification (AUC-ROC) values of the models were low, most likely due to limited variance.

The study has several *strengths and limitations. T*he use of a supervised machine learning approach for variable selection allowed for a comprehensive analysis of numerous clinical factors, providing valuable insights into the prediction of PSP after stroke. The study’s large sample size and multicenter design could enhance the generalizability of the results to a broader stroke population; however, the final regression model was based on 12 variables that could hinder the clinical implications of the results. Variables should have been selected by all ML models with coefficients > 0.01 to be included in the multivariable logistic regression models. This was an important step for achieving the parsimonious models; however, the threshold of > 0.01 could be argued. The study’s observational nature prevents establishing causal relationships between PSP and other factors. Although various statistical techniques were used to handle missing data, the possibility of bias due to missing observations cannot be completely ruled out. We were not able to distinguish between central and peripheral pain syndromes. Additionally, despite efforts to adjust for confounding variables, residual confounding still exists. In particular, we were not able to adjust for preexisting pain, pain intensity measured by the Visual Analogue Scale, as well as prescribed pain medications, as these data were not included in the registry. This gap may affect the interpretation of our findings, and future research should consider including such information for a more comprehensive analysis. We were not able to adjust for the overrepresentation of PSP following thalamic and brainstem strokes [1]. Last, the study’s generalizability might be limited to the specific population and healthcare system in Sweden.

In conclusion, this study demonstrates the complexity of PSP and the usefulness of a supervised machine learning approach in investigating clinical factors associated with PSP after stroke. Important predictors of PSP three months after stroke were region of birth, sex, income, prestroke physical activity, diabetes, neurointerventional therapy, hemiparesis, sensory deficits, and need for assistance before stroke. Patients who reported PSP had a significantly higher one-year mortality rate than those without pain, and those with constant pain had the highest cumulative mortality.

## Electronic supplementary material

Below is the link to the electronic supplementary material.

Supplementary Material 1

## Data Availability

No datasets were generated or analysed during the current study.

## Abbreviations

AIC: Akaike Information Criterion
AUC-ROC: Area Under the Receiver Operating Characteristic Curve
CI: Confidence Interval
COPD: Chronic Obstructive Pulmonary Disease
HR: Hazard Ratio
ICD-10: International Classification of Diseases, 10th Revision
LASSO: Least Absolute Shrinkage and Selection Operator
MICE: Multiple Imputation by Chained Equations
ML: Machine Learning
MoCA: Montreal Cognitive Assessment
NIHSS: National Institutes of Health Stroke Scale
OR: Odds Ratio
PSP: Poststroke Pain
SGPALS: Saltin-Grimby Physical Activity Level Scale
XGBoost: Extreme Gradient Boosting
