# Supplementary material for: Predicting pain and its association with mortality in patients with stroke
Source: BMC Neurol. 2025 Jan 7;25:10. doi: 10.1186/s12883-024-04011-5 (PMC11705892; doi:10.1186/s12883-024-04011-5)
Supplement: Supplementary file 1 — Supplementary Material 1 [file 12883_2024_4011_MOESM1_ESM.docx]

**Supplement**

**Predicting pain and its association with mortality in patients with stroke**

Adam Viktorisson, MD^1,2^; Aref Haj Hashem, MSc^1^; Katharina Stibrant Sunnerhagen, MD, PhD^1,2^; Tamar Abzhandadze, OT, PhD*^1,3^

1. Department of Clinical Neuroscience, Institute of Neuroscience and Physiology, Sahlgrenska Academy, University of Gothenburg, Gothenburg, Sweden
2. Department of Rehabilitation Medicine, Sahlgrenska University Hospital, Gothenburg, Sweden
3. Department of Occupational Therapy and Physiotherapy, Sahlgrenska University Hospital, Gothenburg, Sweden

**Supplementary Table 1.** Drop-out analyses and frequencies of missing observations.

|  | **Included patients**  **n=4160** | **Non-responders n=1280** | **Dead**  **n=1051** | **p-value** | **Missing** |
| --- | --- | --- | --- | --- | --- |
| Age, mean (SD) | 73 (13) | 70 (16) | 82 (10) | <0.001 | 0 / 0 / 0 |
| Female sex | 1941 (47) | 566 (44) | 572 (54) | 0.094 | 0 / 0 / 0 |
| Born in Sweden | 3412 (82) | 864 (68) | 872 (83) | <0.001 | 0 / 0 / 0 |
| Education >12 years | 1037 (25) | 272 (24) | 190 (18) | <0.001 | 74 / 133 / 37 |
| High income | 1529 (37) | 355 (30) | 244 (23) | <0.001 | 15 / 89 / 3 |
| Single household | 1861 (45) | 551 (55) | 621 (61) | <0.001 | 36 / 269 / |
| Smoking | 495 (14) | 174 (18) | 67 (8) | 0.557 | 514 / 295 / 183 |
| Alcohol abuse | 104 (3) | 52 (4) | 27 (3) | 0.042 | 0 / 0 / 0 |
| Prestroke physical activity |  |  |  |  |  |
| Sedentary | 1890 (50) | 607 (59) | 597 (88) | <0.001 | 410 / 245 / 376 |
| Light intensity | 1596 (43) | 384 (37) | 74 (11) |  |  |
| Moderate or high intensity | 264 (7) | 44 (4) | 4 (1) |  |  |
| Prestroke medications |  |  |  |  |  |
| Antihypertensive | 2286 (55) | 540 (53) | 607 (58) | 0.635 | 9 / 262 / 10 |
| Antiplatelet | 1048 (25) | 244 (24) | 324 (31) | 0.048 | 13 / 262 / 15 |
| Anticoagulant | 478 (12) | 119 (12) | 182 (18) | 0.002 | 181 / 278 / 41 |
| Statins | 1135 (27) | 254 (25) | 267 (26) | 0.098 | 12 / 263 / 13 |
| Comorbidities |  |  |  |  |  |
| Atrial fibrillation | 908 (24) | 232 (23) | 403 (44) | <0.001 | 417 / 270 / 124 |
| Cancer | 453 (11) | 99 (8) | 178 (17) | 0.224 | 0 / 0 / 0 |
| Depression | 218 (5) | 89 (7) | 58 (6) | 0.083 | 0 / 0 / 0 |
| Diabetes | 803 (19) | 291 (23) | 215 (21) | 0.022 | 0 / 0 / 0 |
| COPD | 326 (8) | 97 (8) | 109 (10) | 0.158 | 0 / 0 / 0 |
| Renal failure | 298 (7) | 104 (8) | 104 (10) | 0.011 | 0 / 0 / 0 |
| Hyperlipidemia | 688 (17) | 201 (16) | 145 (14) | 0.079 | 0 / 0 / 0 |
| Prior stroke | 634 (15) | 153 (15) | 234 (23) | <0.001 | 10 / 262 / 11 |
| Stroke type |  |  |  |  |  |
| Ischemic stroke | 3761 (91) | 885 (87) | 832 (79) | <0.001 | 8 / 258 / 4 |
| Intracerebral hemorrhage | 391 (9) | 137 (13) | 215 (21) |  |  |
| Interventional therapy |  |  |  |  |  |
| Neurosurgery | 17 (1) | 15 (1) | 11 (1) | 0.009 | 10 / 232 / 5 |
| Thrombectomy | 464 (12) | 124 (14) | 207 (24) |  | 293 / 367 / 169 |
| Thrombolysis | 614 (15) | 134 (13) | 149 (14) |  | 11 / 263 / 3 |
| NIHSS, mean (SD) | 5 (6) | 6 (7) | 13 (8) | <0.001 | 231 / 150 / 165 |
| Hemiparesis | 1181 (34) | 352 (40) | 385 (71) | <0.001 | 655 / 391 / 505 |
| Sensory deficit | 932 (27) | 268 (31) | 266 (52) | <0.001 | 722 / 415 / 542 |
| Cognition* |  |  |  |  |  |
| Normal | 698 (21) | 95 (10) | 8 (2) | <0.001 | 815 / 328 / 548 |
| Impaired | 1247 (37) | 333 (35) | 170 (39) |  |  |
| Unclear | 1400 (42) | 524 (55) | 325 (65) |  |  |
| Need of assistance | 683 (17) | 245 (25) | 450 (48) | <0.001 | 211 / 287 / 117 |
| Values are presented as No. (%) unless otherwise stated. Abbreviations: COPD, Chronic obstructive pulmonary disease; NIHSS, National Institutes of Health Stroke Scale. Group comparisons were performed using the χ² test for categorical variables and the Mann Whitney U-test for continuous variables. For these analyses, non-respondents and deceased patients were combined (n=2331).  *Cognition was evaluated during the hospital stay using the Montreal Cognitive Assessment (MoCA), which could not be conducted in 2249 patients due to aphasia (n=411), lack of a translator for foreign language speakers (n=307), severe impairments such as diplopia, hearing loss, fatigue, or worse general condition (n=803), death before assessment (n=89), patient's declination to participate (n=88), or logistic errors (n=551). | | | | | |

**
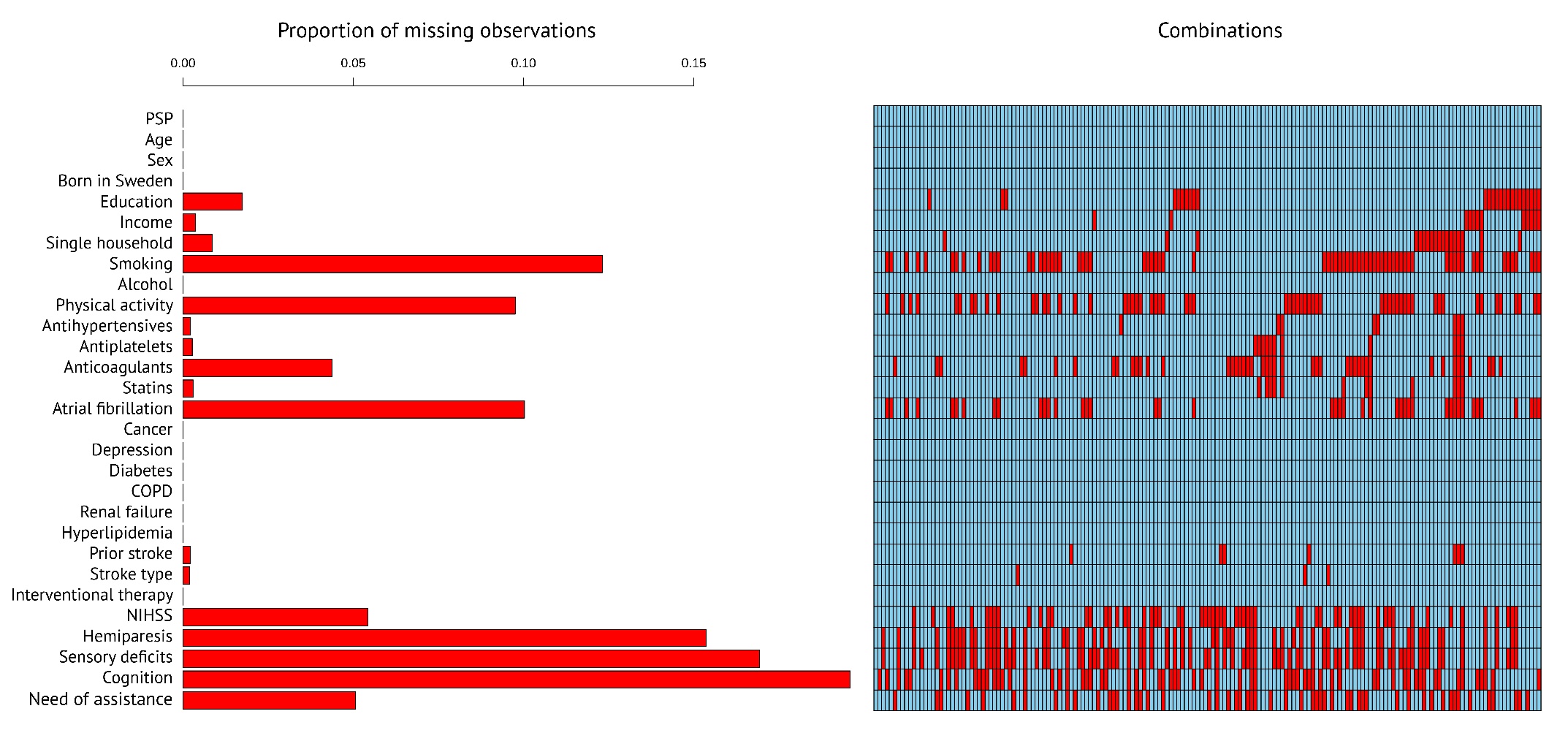
Supplementary Figure 1.** Missing data matrix.

Abbreviations: COPD, Chronic obstructive pulmonary disease; NIHSS, National Institutes of Health Stroke Scale.

**
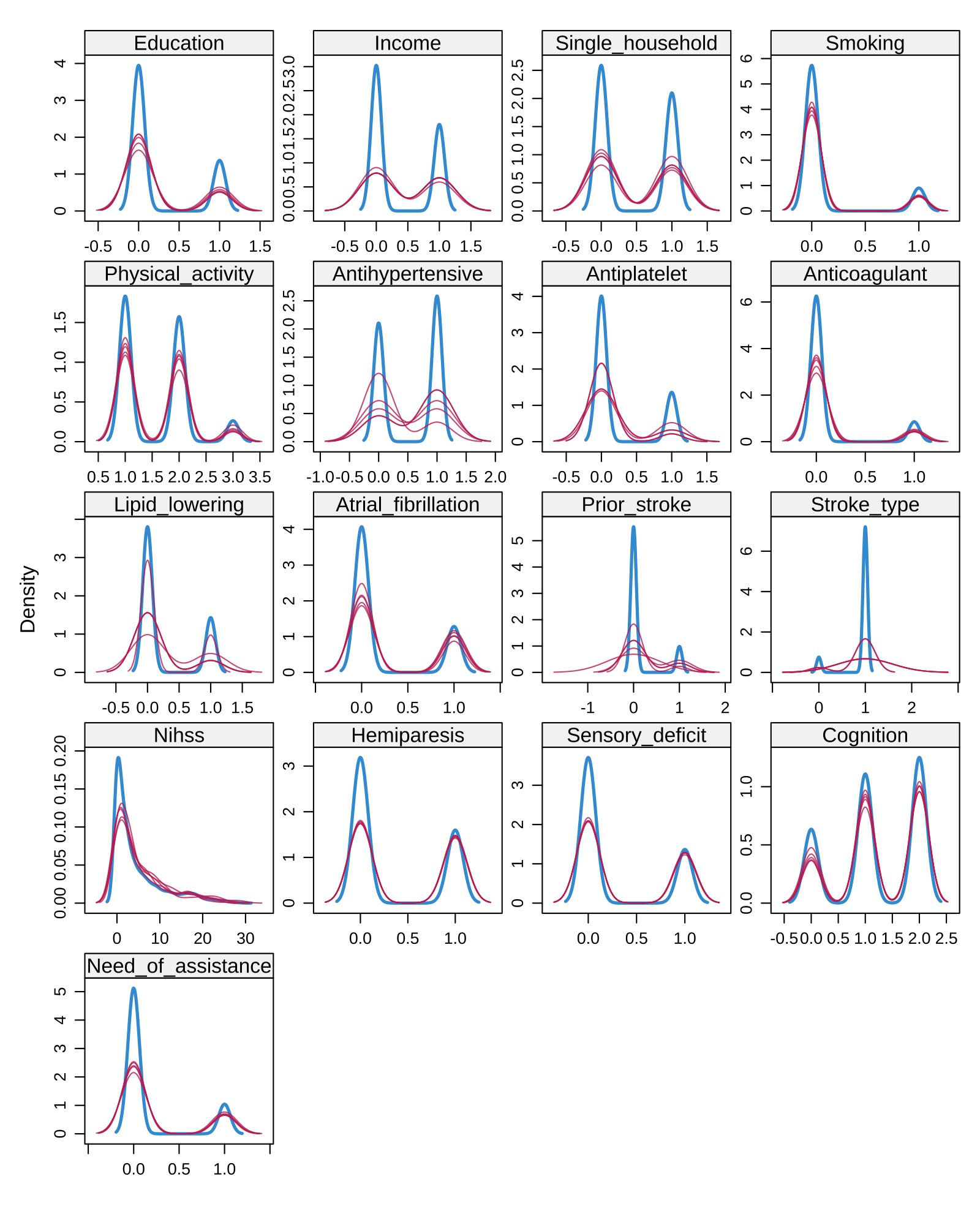
**

**Supplementary Figure 2.** Density plot for imputed and observed values in variables with missing data.

Multiple imputation by chained equations (MICE) was used to handle missing observations, with five imputed datasets per variable. The imputation process was repeated with up to 20 iterations for each imputed dataset. The density plots show the distribution of imputed values compared to the observed values for each variable in the dataset. The density plots of the imputed values (shown in red lines) match the density plots of the observed values (blue lines), which indicates that the imputed values are reasonable and similar to the actual data.
